# Supplementary material for: Simulation of Soluble and Bound VEGF-stimulated in vitro Capillary-like Network Formation on Deformed Substrate
Source: PLoS Comput Biol. 2024 Jul 22;20(7):e1012281. doi: 10.1371/journal.pcbi.1012281 (PMC11262697; doi:10.1371/journal.pcbi.1012281)
Supplement: S1 Appendix — (DOCX) [file pcbi.1012281.s001.docx]

**Appendix. Friction Force Exerted by the Petri Dish**

During the *in vitro* cultivation of capillary plexus, the inertia forces and gravity in biogel are negligeble compared to viscous and elastic effects [1, 2]. Considering the cell-free case, the force balance in the biogel yields the following equation, which conforms to the summation convention over the repeated subindices.

$\nabla\cdot\mathbf{\sigma}=\frac{\partial}{\partial x}\mathbf{\sigma}_{xi}\hat{e}_{i}+\frac{\partial}{\partial y}\mathbf{\sigma}_{yi}\hat{e}_{i}+\frac{\partial}{\partial z}\mathbf{\sigma}_{zi}\hat{e}_{i}=0$ (A1)

where the bold fonts represent the second-order tensors. We take $i=x$ and $y$ for the 2-D simplication in this work. After integrating from the biogel bottom ($z=0$) to the top surface ($z=h(x,y)$), Equation (A1) reads:

$\int_{0}^{h} \frac{\partial}{\partial x}\mathbf{\sigma}_{xi}dz+\int_{0}^{h} \frac{\partial}{\partial y}\mathbf{\sigma}_{yi}dz+\int_{0}^{h} \frac{\partial}{\partial z}\mathbf{\sigma}_{zi}dz=0$, $i=x,\text{ }y$. (A2)

The first term in Eq. (A2) can be expanded based on the Leibniz rule and rearranged as follows:

$\begin{matrix} \int_{0}^{h} \frac{\partial}{\partial x}\mathbf{\sigma}_{xi}dz=\frac{\partial}{\partial x}\int_{0}^{h} \mathbf{\sigma}_{xi}dz-\frac{\partial h}{\partial x}\left.\mathbf{\sigma}_{xi}\right|_{z=h} \\ =\frac{\partial}{\partial x}\left(h\bar{\mathbf{\sigma}}_{xi}\right)-\frac{\partial h}{\partial x}\left.\mathbf{\sigma}_{xi}\right|_{z=h} \\ =\frac{\partial h}{\partial x}\bar{\mathbf{\sigma}}_{xi}+h\frac{\partial}{\partial x}\bar{\mathbf{\sigma}}_{xi}-\frac{\partial h}{\partial x}\left.\mathbf{\sigma}_{xi}\right|_{z=h} \\ \approx h\frac{\partial}{\partial x}\mathbf{\sigma}_{xi} \end{matrix}$ (A3)

where the averaged stress across the biogel thickness is denoted as $\bar{\mathbf{\sigma}}_{xi}$, and a uniform traction force on the longitudinal plane $\bar{\mathbf{\sigma}}_{xi}=\mathbf{\sigma}_{xi}$ is presumed. Similarly, the second term in Eq. (A2) can be simplified as

$\int_{0}^{h} \frac{\partial}{\partial y}\mathbf{\sigma}_{yi}dz\approx h\frac{\partial}{\partial y}\mathbf{\sigma}_{yi}$ (A4)

Finally, integrating the third term in Eq. (A2) yields

$\begin{matrix} \int_{0}^{h} \frac{\partial}{\partial z}\mathbf{\sigma}_{zi}dz=\left.\mathbf{\sigma}_{zi}\right|_{z=0}^{z=h} \\ =\left.\mathbf{\sigma}_{zi}\right|_{z=h}-\left.\mathbf{\sigma}_{zi}\right|_{z=0} \end{matrix}$ (A5)

Rewriting Eq. (A2), substituting from Eq. (A3)-(A5), and dividing with the biogel thickness $h$ yields the thickness-averaged stress balance equation

$\frac{\partial}{\partial x}\mathbf{\sigma}_{xi}+\frac{\partial}{\partial y}\mathbf{\sigma}_{yi}+\frac{1}{h}\left.\mathbf{\sigma}_{zi}\right|_{z=h}=\frac{1}{h}\left.\mathbf{\sigma}_{zi}\right|_{z=0}$, $i=x,\text{ }y$. (A6)

Since there is no external force at the surface $z=h$, the last term on the left-hand side of Eq. (A6) can be regarded as zero

$\frac{1}{h}\left.\mathbf{\sigma}_{zi}\right|_{z=h}=0$ (A7)

On the other hand, the shear stress at the gel bottom $z=0$ is a combination of viscous, linear elastic, and long-range elastic forces

$\left.\mathbf{\sigma}_{zi}\right|_{z=0}=\frac{E}{1+\nu}\left.\mathbf{\varepsilon}_{zi}\right|_{z=0}-\frac{E}{1+\nu}\beta_{1}\nabla^{2}\left.\mathbf{\varepsilon}_{zi}\right|_{z=0}+\mu_{1}\frac{\partial}{\partial t}\left.\mathbf{\varepsilon}_{zi}\right|_{z=0}$ (A8)

where the forms are similar to Eq. (13)-(15) but with the gel dilatation $\theta=\nabla\cdot\mathbf{u}$ equal to zero here because of the no-slip biogel attachment on the Petri dish. By further simplifying the thin gel strain $\left.\mathbf{\varepsilon}_{zi}\right|_{z=0}\approx\frac{u_{i}}{h}$, Equation (A6) takes the form

$\frac{\partial}{\partial x}\mathbf{\sigma}_{xi}+\frac{\partial}{\partial y}\mathbf{\sigma}_{yi}=\frac{1}{h^{2}}\left(\frac{E}{1+\nu}u_{i}-\frac{E}{1+\nu}\beta_{1}\nabla^{2}u_{i}+\mu_{1}\frac{\partial u_{i}}{\partial t}\right)$, $i=x,\text{ }y$ (A9)

The right-hand side of Eq. (A9) accounts for the friction force exerted by the Petri dish.

$\mathbf{R}_{ext}=\frac{1}{h^{2}}\left(\frac{E}{1+\nu}\mathbf{u}-\frac{E}{1+\nu}\beta_{1}\nabla^{2}\mathbf{u}+\mu_{1}\frac{\partial\mathbf{u}}{\partial t}\right)$ (A10)

**References**

1. Manoussaki D, Lubkin S, Vemon R, Murray J. A mechanical model for the formation of vascular networks in vitro. Acta biotheoretica. 1996;44:271-82.
2. Namy P, Ohayon J, Tracqui P. Critical conditions for pattern formation and in vitro tubulogenesis driven by cellular traction fields. Journal of Theoretical Biology. 2004;227(1):103-20.
