# Supplementary material for: Simulation of Soluble and Bound VEGF-stimulated in vitro Capillary-like Network Formation on Deformed Substrate
Source: PLoS Comput Biol. 2024 Jul 22;20(7):e1012281. doi: 10.1371/journal.pcbi.1012281 (PMC11262697; doi:10.1371/journal.pcbi.1012281)
Supplement: Attachment — Submitted filename: Responses to Reviewer3 0409.docx [file pcbi.1012281.s002.docx]

Reviewer #3

We thank the Reviewer for critically reviewing the manuscript and for the valuable suggestions to improve its quality. The constructive feedback on the manuscript has helped improve the contents. We have revised the manuscript per the Reviewer's comments. All the major and minor suggestions made by the Reviewer have been incorporated in the revised manuscript. Please find below the point-wise responses to the comments.

Major comments:

=> line 171. The authors introduce the probability for the cell to become elongated per unit time. Does it become elongated permanently? If the cell moves to a region with a low VEGF concentration, or if it becomes surrounded by other cells, can it transform back to the flattened phenotype? This dynamics of phenotype change is not well explained in the document. Why is the value of \mu_m so large? It seems that immediately that the cell can, it will change to its elongated phenotype. Also, though I understand the reason for introducing r_sigma, so that when a cell is surrounded by other cells it does not become elongated, it is very hard to understand from the biological point of view. A cell should have VEGF receptors on all its membrane surface, including the part above (the apical region), and the soluble VEGF should bind those receptors, not only the receptors at the perimeter of the cell. Therefore, the presence of neighbouring cells should not affect significantly the ability for a cell to bind soluble VEGF when it is plated in a flat substrate.

Following the cell morphology classification (Stamati et al., 2014), we consider cell elongation (with cytoskeletal solidification and cytoplasm merging) irreversible differentiation [reference 46]. We have strengthened the description in Section 5.1, page 32, line 566.

We estimated the mu_m value based on the ratio of elongated cells observed in the reference experiments: approximately 33% of the cells become elongated after 12 hours of HUVEC culturing. Therefore, we set the same VEGF concentration as the experiment and fitted the mu_m value to have 33% of cells in the simulation reach the morphologized label after 12 hours. The explanation on determining cell-morphology parameter values has been added as in red on page 38.

Aggregated cell clusters maintain cobblestone-like characteristics, as observed in previous experiments (Vailhé et al.,1997, Serini et al., 2003, and Stamati et al., 2014) [references 7, 45, 46]. Based on the experimental observation, we thus suppressed the cell elongation if it was surrounded by other cells in the simulation. Detailed mechanisms behind this phenomenon require further investigation. We have added discussion and explained that the biological mechanism behind the cell surrounding cue requires further investigation, as in red on page 26, lines 447 – 450.

=> line 215. Here, the stress is approximately constant throughout the cell. That is a different approach from van Oers et al 2014, and from the experimental measures of traction force microscopy by Reinhart-King group, where the traction force increases from the center to the periphery of the cell. Why did the authors decided to implement a stress that is approximately constant inside the cell?

Since we study the biogel deformation by cell traction and cell morphology change by the VEGF signaling, we have neglected the stress gradient effects inside the cells. We have cited van Oers et al. and added a discussion on the limitation of our model, as denoted in red on page 27. Thank you.

=> lines 249, 250. The authors should justify why VEGF intake by the endothelial cells is not considered.

We aimed to explore the endothelial network initiation induced by the paracrine signaling for short-term cultivation. Thus, we consider the effects of VEGF added in the culture medium and neglect the VEGF secretion from cells and intake by cells. We have clarified this simplification in Section 5.3, as in red on page 36

=> The process of choosing the parameters is not conveniently explained:
=>=> MCS is chosen as 2 minutes so that the cells migrate at a "reasonable speed". What is a reasonable speed? Did the authors carry out migration simulations of isolated cells? How was the comparison with experiment carried out?

We estimated the cell's moving speed by calculating the moving distance of its geometric center within a period. By setting 1 MCS as 2 minutes in our simulation, the maximum moving distance of cells was about 30 μm for 30 MCS. This maximum moving speed of cells is consistent with the measurement by Shiu et al. (2004), about 30 μm/hr [reference 63]. We have added an explanation on page 37, line 666.

=>=> mu_m was determined to have approximately one third of elongated cells? Why one third? How different is the number of elongated cells in the simulation and in the experiment?

The previous experiment (Chiang and Chung, 2021) showed that one-third of seeded cells elongated after 12 hours of incubation [reference 44]. By fitting the simulation with the experimental results, we determined the mu_m value to have one-third of cells elongated after 360 MCS, corresponding to 12 hours of incubation. We have added an explanation on page 38, lines 680-682.

=>=> The Cellular Potts parameters were chosen based on the average area and length of the cells. What are the values and how do they compare to experiments?

The previous experiment (Chiang and Chung, 2021) showed that the diameter of round cells was about 50 μm, and the cell area was about 1960 μm^2 [reference 44]. We fitted the cell area and perimeter to determine the area and perimeter restraint parameters. We determined the cell length restraint parameter by fitting the elongated cells that reached about 2.5 times the initial diameter in simulation as observed in the experiment. We have added an explanation on page 38, lines 682-685.

=>=> The amount of fibronectin was chosen so that there was a reasonable amount of bound VEGF. What is a reasonable amount? How did the authors compare to experiments?

For the amount of fibronectin, we set its value so that there was approximately 10% of the added VEGF transferred from soluble to bound form, which fitted the measurement of Goerges (2004) considering fibronectin-coated substrates in a weakly acidic environment [reference 64]. We have added an explanation on page 38, line 688.

=> line 399. The authors indicate that the results observed in figure 7 match the observations for elongated cells. And for the flattened cells? Why does the model do not reproduce their movement? Maybe this part needs more clarification by describing in greater detail how the comparison between the experimental and simulation results was carried out.

We have deleted this misleading sentence and corrected it into "The results agreed with the experiment observation for the elongated cells [references 7, 54, 55]", as line 294 on page 17.

=> Figures 8 and 9. Here, the authors measure the number of junctions until it saturates, as a function of different parameters. However, it is nor clear for which number of junctions functional networks are obtained. If the number of junctions is large I presume that the cells are clustered together. The authors could analyse the different networks to identify the interval of number of junctions that correspond to networks, as it was done in Ramos et al, Phys Rev E 2018.

We have added cell distribution diagrams in Figure 9 to identify the range for junction numbers. The results show that the cell distribution gradually evolved into a stable network with no open loops. The junction number approached a corresponding asymptotic value ranging from 160 to 200 for 1600 cells on the substrate of 11.56 mm^2 in the sight.

=> In figures 8 and 9 what are the size of the error bars? I.e. what variation can be expected in the number of junctions when running several simulations with the same parameters? Also, in figure 9 it is shown a prediction for the number of junctions for different values of VEGF concentration, c0. That experiment can be done in the lab. Does the simulation match the experimental results?

Ex-Figures 8 and 9 (Figures 9 and 10 of the revised version) aim to investigate how parameters influence network development. Thus, we conducted one CPM simulation for the same parameters, considering that the standard deviation of junction number is about 8% when running three simulations with the same typical parameter values, as shown in Figure 4 (Ex-Figure 6). We believe the data trend and the regression curves will be similar for different CPM simulations with the same parameters. We have no experiments regarding different VEGF concentrations, which is worth future research. In our knowledge, the excessive dosage of VEGF may result in pathological blood vessels, whereas insufficient VEGF would decrease the activities and survival rate of endothelial cells.

=> Besides the issues of the soluble VEGF binding in the apical region of the cell, I find the dynamics of bound VEGF problematic. In the simulation, bound VEGF concentration is larger underneath the cells. But how does it end up there? The diffusion of soluble VEGF through the matrix is hampered as the matrix is a porous media and also because of the VEGF binding sites. Therefore, by which mechanism does the soluble VEGF reach the underneath of the cell in such high concentration to bind? Moreover, there are three mechanisms that are not simulated that can alter this picture: secretion of VEGF by endothelial cells, intake of VEGF by endothelial cells, and production of MMPs that release VEGF from the matrix. Is it observed experimentally higher levels of bound VEGF underneath the cell? And if so, does that VEGF come from secretion of endothelial cells or from the soluble VEGF that finds more binding sites underneath the cell?

We consider that soluble VEGF is binding onto the substrate via fibronectin, and then the bound VEGF is pulled by cells' traction along with the substrate's VEGF binding sites. Because of this mechanism, the bound VEGF concentration is more prominent at the cell sites. The diffusion of soluble VEGF through the matrix pores and the transport hampered by the cells were neglected.

The three mechanisms, secretion of VEGF by endothelial cells, intake of VEGF by endothelial cells, and production of MMPs that release VEGF from the matrix, were not included in the present model because we focused on the cell network initiation induced by paracrine signaling for short-term cultivation within a day. The culture medium offers the necessary chemicals during the capillary development process. We emphasize this consideration in the Introduction on pages 3 and 8, as denoted in red. Thank you.

=> Line 544. Here, the authors mention that purely mechanical regulation is insufficient to model cell orientation and network formation. However, in van Oers et al 2014 the authors suggest a model that uses only mechanics to model similar mechanisms as in the present paper. From what I understand, here the simulated mechanism is the following: cell traction increases cell density under the cell, that leads to more binding of VEGF in the matrix, which drives the hapoptaxis and alignment of the cells which the soluble VEGF made elongated. In van Oers et al 2014 the forces elongate the cells and the cells migrate in the direction of larger strain, i.e. in the direction of other cells, with a similar effect of the VEGF haptotaxis and without the mechanism of having large concentrations of bound VEGF under the cells, and without the mechanism of requiring soluble VEGF to elongate the cells. Therefore, I would argue that in principle, a purely mechanical simulation could reproduce the same observations as in this simulation, with a particular choice of parameters. Could the authors better justify this point?

We have deleted this misleading sentence and included van Oers et al. (2014) [reference 37] in the literature review and the Discussion sections. Because not all attached-type cells aggregated and elongated on substrates, we thought there might not be only a single mechanism that guides the cell migration and induces the cell aggregation patterns. The model we suggested in the present study may be one of them, as it relates to the endothelial cell line. We have added a discussion citing van Oers et al. (2014) on page 27.

=> Line 565. The authors indicate that this model could be used for sprouting angiogenesis if proliferation was included. Did the authors simulate the evolution of a circular cell colony to see if sprouts are able to extend using this model? What is the sprout width in those cases? In sprouting angiogenesis, cell specialisation into tip and stalk cells is essential. Therefore I would think that, besides proliferation, these different phenotypes would have been to be included.

We have deleted this inappropriate indication. In sprouting angiogenesis, cell specialization into tip and stalk cells should be essentially considered.

Minor Points

=> table 1. The mu_m should have units um^3/ng/mcs, as it defines the probability per Montecarlo step, correct?

We have checked the parameter units. Because Eq. (9) represents the probability of cells experiencing morphological change, the combined parameter group is dimensionless, and the units for mu_m as μm^3/ng are correct. Equation 8 regarding displacement probability per Montecarlo step is the other probability function in the CPM, which is dimensionless, too.

=> line 199. A technical detail: when the simulation is run, in each spin flip trial, does the code choose any point in the lattice (in a way that it can choose points inside a cell or inside the ECM, leading to many flip trials that are not successful because the spin is surrounded by spins of the same cell), or, in the other hand, does the code keep a list of cell boundary points and only chooses a point to flip from that list? If the latter happens, the number of flips in a MCS is less than the number of lattice sites. Could the authors clarify?

The randomly selected lattice is independent of the current state, whether it is occupied by cell or cell-free. Whether the chosen lattice can transfer its state to its neighbor follows the probability determined by the whole system's Hamiltonian change. We have clarified it on page 33, lines 587-590.

=> Throughout the text the authors mention "parametric values". They should mention "parameters", or "simulation parameters" instead.

We have changed the wording. Thank you.

=> Images are too small in figures 4, 6, 7, 8, 9, and 10. The numbers and legends are not clear. Consider having the image occupy more than half a page.

We have revised the figures and hope they will be easy to read. Thank you.
