# Supplementary material for: Simulation of Soluble and Bound VEGF-stimulated in vitro Capillary-like Network Formation on Deformed Substrate
Source: PLoS Comput Biol. 2024 Jul 22;20(7):e1012281. doi: 10.1371/journal.pcbi.1012281 (PMC11262697; doi:10.1371/journal.pcbi.1012281)
Supplement: Attachment — Submitted filename: Responses to Reviewer1 0611.docx [file pcbi.1012281.s003.docx]

Reviewer #1:

We thank the Reviewer for the valuable suggestions. We have revised the manuscript per the Reviewer's comments. Please find below the point-wise responses.

[1] In response to Rev 1 (E):

"Since cell culturing in incubators is at 37°C, our model does not include temperature as a variable. .. It will affect the cell movement and deformation, including shape and size."

And correction on L 583-584 of the paper.

This is a total misunderstanding by the authors of the CPM temperature parameter. This parameter has nothing to do with the lab temperature. It is just a quantity in CPM that controls the magnitude of fluctuations. It's OK to say "we kept the temperature parameter constant, T=1", but the lab temperature has nothing to do with it.

We have revised the sentences following your comments, as denoted in red on page 34, lines 599-601. Thank you.

[2] More detailed figure captions were requested in the review. The revised paper makes it hard to find those captions, which are scattered between lines of text.

The layout of the figure captions followed the journal guidelines, which request that the figure captions appear after the paragraph where they are first cited. We have denoted the figure captions in blue to make it clear.

[3] The supplementary file is not in a very good shape. There are some weird notes like "MERGEFORMAT" right in the middle of the equations. Also, the equations are not fully readable. To be more specific:

--> In equation (A2) it is hard to see the limits of the integral,

--> In equation (A3) it is quite hard to read the subscripts,

--> In equation (A8), in the Laplacian operator (nabla to the power 2): the power 2 is not very visible.

We have adjusted all the subscript sizes throughout the S1 Appendix. We also uploaded the PDF file to avoid conversion errors. Thank you.
